# Increase in esodeviation under cycloplegia with 0.5% tropicamide and 0.5% phenylephrine mixed eye drops in patients with hyperopia and esotropia

**DOI:** 10.1186/s12886-017-0644-7

**Published:** 2017-12-12

**Authors:** In Jeong Lyu, Kyung-Ah Park, Sei Yeul Oh

**Affiliations:** 1 Department of Ophthalmology, Samsung Medical Center, Sungkyunkwan University School of Medicine, Seoul, Korea; 2 Department of Ophthalmology, Nowon Eulji Medical Center, Eulji University School of Medicine, Seoul, Korea

**Keywords:** Esotropia, Hyperopia, Accommodative esotropia, Cycloplegia

## Abstract

**Backgroud:**

To evaluate the manifestations of increased esodeviation under cycloplegia with 0.5% tropicamide and 0.5% phenylephrine in children with hyperopia and esotropia.

**Methods:**

We reviewed the medical record of 34 children with hyperopia and esotropia who underwent a prism alternate cover test before and after instillation of mixed eye drops containing 0.5% tropicamide and 0.5% phenylephrine between November 2014 and October 2015. Increased angle of deviation was defined as 10 prism diopters (PD) or greater deviation after cycloplegia. The factors related to increased angle of deviation were evaluated using univariable and multivariable logistic regression analysis.

**Results:**

The median age was 5.0 years (interquartile range, 3.75 to 5.0) and 12 patients (35.3%) were male. The median manifested refractive (MR) was +2.13 diopters (D) (+0.92 to +4.47) and cycloplegic refractive (CR) was +3.50 D (+1.72 to +5.66). The median difference between MR and CR was +0.88 D (+0.50 to +1.28). Thirteen patients (38.2%) showed increased esodeviation under cycloplegia and all had accommodative esotropia. A larger difference between MR and CR was the only significant factor affecting increased esodeviation in both univariable (OR = 4.72, *P* = 0.029) and multivariable (OR = 5.22, *P* = 0.047) analyses.

**Conclusion:**

Children with hyperopia and esotropia often showed an increased angle of deviation after instillation of 0.5% tropicamide and 0.5% phenylephrine. This phenomenon reminded the clinicians that cycloplegics can have a different effect on esodeviation and suggested that increased angle of esodeviation may help to reveal the latent deviation in some patients with hyperopia and esotropia.

## Background

Cycloplegic refraction (CR) is a crucial procedure for strabismus evaluation, especially in patients with esotropia, because that enables pediatric patients to display the full amount of hyperopia by preventing accommodation. Traditionally, atropine sulfate has been used for cycloplegia. However, atropine requires long periods of time to obtain a maximal cycloplegic effect and recovery from cycloplegia is much longer than the cycloplegic effect. It can also induce systemic side effects [1]. Therefore, 1% cyclopentolate, 1% tropicamide, or mixed eye drops containing 0.5% tropicamide and 0.5% phenylephrine are widely used because of their safety, rapid onset, and quick recovery [2–5]. However, we observed that ocular deviation was increased after instillation of combination drops containing 0.5% tropicamide and 0.5% phenylephrine in some patients with esotropia and hyperopia. Herein we reported and evaluated the increased esodeviation in patients with hyperopia and esotropia under cycloplegia.

## Methods

This study was performed in accordance with the tenets of the Declaration of Helsinki. Approval to conduct this study was obtained from the Institutional Review Board of Samsung Medical Center (Seoul, Republic of Korea). Children with hyperopia and esotropia who underwent a prism alternate cover test before and after cycloplegia on the same day at Samsung Medical Center between November 2014 and October 2015 were retrospectively reviewed. Patients with history of strabismus surgery, retinopathy of prematurity, intraocular surgery, paralytic or restrictive strabismus, neurologic disorders, congenital anomaly, or other ophthalmic or systemic diseases were excluded.

Comprehensive ophthalmic examination was performed in all patients by a single pediatric ophthalmologist (S.Y.O.). Manifested refraction (MR) using retinoscopy and best corrected visual acuity (BCVA) were measured. Amblyopia was defined as the inter-ocular difference in BCVA of ≥2 lines (logarithm of the minimal angle of resolution [logMAR]). In five preverbal children (14.7%) whose BCVA was not measurable, we measured MR in the dark room while the children were fixating at lighting animated toys at the distance (6 m). A 10-diopter prism-down fixation test was also performed to detect amblyopia in preverbal patients.

Ocular alignment was tested by a prism alternate cover test at 6 m and 33 cm fixation. Combination drops containing 0.5% tropicamide and 0.5% phenylephrine (Mydrin-P, Santen, Osaka, Japan) was administered to both eyes in three doses separated by 5 min. Refraction and a prism alternate cover test under cycloplegia were repeated 30 min after administration of the first eye drops. We defined increased angle of esodeviation as 10 prism diopters (PD) or greater deviation under cycloplegia. Fundus examination was also performed to examine accompanying ophthalmic diseases. The spherical equivalent was calculated as the sphere plus half a cylinder. Spectacles were prescribed based on CR and the entire refractive error was fully corrected.

A child was diagnosed with fully refractive accommodative esotropia (FAET) if the deviation was corrected less than 10 PD with correction, partially refractive accommodative esotropia (PAET) if there was reduction of the deviation with spectacles but there was residual esotropia of 10 PD or more, and non-accommodative esotropia (NAET) if spectacle correction did not have the effect on the deviation. Accommodative ET with a high AC/A ratio was diagnosed when the near esodeviation exceeded the distance measurement by 10 PD or more with spectacle correction and if it decreased at near with additional plus 3.0 D lenses [6].

Statistical analysis was performed using the commercially available statistical package SPSS ver. 18.0 for Windows (SPSS Inc., Chicago, IL, USA). Continuous data are presented as medians with interquartile range (IQR), and categorical data are presented as counts with percentages. Univariable and multivariable logistic regression analyses were performed to investigate factors associated with increased esodeviation. Analysis was first performed using an univariable model, and the multivariable model adjusted for age, gender, MR, and angle of deviation.

## Results

### Baseline characteristics

We identified a total of 40 children with hyperopia and esotropia who underwent ocular alignment by prism alternate cover testing before and after cycloplegia on the same day. Among these patients, six were excluded from the study for the following reasons: chromosomal anomaly (*n* = 2); cerebral palsy or developmental delay (*n* = 3); and history of retinopathy of prematurity (*n* = 1). A total of 34 patients were included in this study.

The median age was 5.0 years (IQR 3.75 to 5.0) and 12 patients (35.3%) were male. Twenty-three patients (67.6%) had FAET, seven patients (20.6%) had PAET, one patient (2.9%) had accommodative ET with a high AC/A ratio, and three patients (8.8%) had NAET. Amblyopia was diagnosed in seven patients (20.6%). The median MR was +2.13 D (IQR +0.92 to +4.47) and CR was +3.50 D (+1.72 to +5.66). The median difference between MR and CR was +0.88 D (+0.50 to +1.28). Other demographic data are summarized in Table 1.

**Table 1:** Demographics of patients with and without angle change after cycloplegia

Variables, Median (IQR)	Increased angle of deviation (*n* = 13)	No change in deviation (*n* = 21)	Total (*n* = 34)
Age (years)	5.0 (4.0, 5.0)	5.0 (4.0, 5.0)	5.0 (4.0, 5.0)
Gender (male:female)	4:9	8:13	12:22
Type of esotropia			
FAET	8 (61.5%)	15 (71.4%)	23 (67.6%)
PAET	4 (30.8%)	3 (14.3%)	7 (20.6%)
High AC/A ratio	1 (7.7%)	0 (0.0%)	1 (2.9%)
NAET	0 (0.0%)	3 (14.3%)	3 (8.8%)
Presence of amblyopia	3 (23.1%)	4 (19.0%)	7 (20.6%)
Manifested SE refractive error (diopters)	+1.56 (+0.57, +3.50)	+2.13 (+1.32, +5.88)	+2.13 (+0.92, +4.47)
Cycloplegic SE refractive error (diopters)	+3.75 (+1.88, +4.25)	+3.25 (+1.60, +6.44)	+3.50 (+1.72, +5.66)
Cycloplegic-manifested refractive error (diopters)	+1.13 (+0.53, +1.88)	+0.63 (+0.44, +1.16)	+0.88 (+0.50, +1.28)
Manifested angle of deviation at far distance (PD)	15.0 (10.0, 35.0)	15.0 (10.0, 30.0)	15.0 (10.0, 35.0)
Manifested angle of deviation at near distance (PD)	15.0 (10.0, 35.0)	15.0 (10.0, 30.0)	15.0 (10.0, 35.0)
Cycloplegic angle of deviation at far distance (PD)	30.0 (20.0, 45.0)	15.0 (10.0, 30.0)	21.0 (12.0, 40.0)
Cycloplegic angle of deviation at near distance (PD)	35.0 (25.0, 45.0)	20.0 (12.0, 30.0)	25.0 (15.0, 40.0)
Increase in esodeviation at far distance (PD)	10.0 (10.0, 10.0)	0.0 (0.0, 2.0)	2.5 (0.0, 10.0)
Increase in esodeviation at near distance (PD)	10.0 (10.0, 20.0)	2.0 (0.0, 5.0)	5.5 (0.0, 10.0)

*FAET* fully refractive accommodative esotropia, *PAET* partially refractive accommodative esotropia, *NAET* non-accommodative esotropia, *SE* spherical equivalent, *PD* prism diopters

### Changes in angle of deviation before and after cycloplegia

The median angle of esodeviation before cycloplegia was 15 PD (IQR 10 to 35) at far distance and 15 PD (IQR 10 to 35) at near distance. The median angle of deviation after cycloplegia was 21 PD (IQR 12 to 40) at far distance and 25 PD (IQR 15 to 40) at near distance. None of the patients showed decreased esodeviation after cycloplegia compared to before cycloplegia, whereas 13 patients (38.2%) showed increased esodeviation, including eight patients with FAET, four patients with PAET, and one with accommodative ET with a high AC/A ratio. In these patients, the median angle of deviation before cycloplegia was 15 PD (IQR 10 to 35) at far distance and 15 PD (IQR 10 to 35) at near distance. The median angle of deviation after cycloplegia was 30 PD (IQR 20 to 45) at far distance and 35 PD (IQR 25 to 45) at near distance. The median increment of esodeviation was 10 PD (IQR 10 to 10) and 10 PD (IQR 10 to 20) at far and near distance, respectively (Table 1).

### Factors influencing increased esodeviation under cycloplegia

Univariable and multivariable logistic regression analysis were used to detect factors influencing increased esodeviation (Table 2). The difference between MR and CR was the only significant factor affecting increased esodeviation in both univariable (OR = 4.72, *P* = 0.029) and multivariable analyses (OR = 5.22, *P* = 0.047).

**Table 2:** Factors affecting increased esodeviation under cycloplegia

Variables	Univariable model			Multivariable model^a^		
	OR	95% CI	*P*- Value	OR	95% CI	*P*- Value
Age (years)	0.993	0.605–1.628	0.976			
Female gender	1.385	0.318–6.026	0.665			
Manifested SE refractive error (diopters)	0.735	0.504–1.072	0.110			
Cycloplegic SE refractive error (diopters)	0.852	0.594–1.222	0.385			
Cycloplegic-manifested refractive error (diopters)	4.719	1.176–18.932	0.029*	5.224	1.019–26.788	0.047*
Manifested angle of deviation at far distance (PD)	1.012	0.972–1.053	0.574			
Manifested angle of deviation at near distance (PD)	1.012	0.971–1.055	0.572			
Presence of amblyopia	1.594	0.286–8.871	0.595			
Presence of refractive accommodative component	0.000	0.000–0.000	0.999			

*OR* odds ratio, *CI* confidence interval, *SE* spherical equivalent, *PD* prism diopters

^a^Adjusted for age, sex, manifested refractive error, manifested angle of deviation at far and near distance

*P-values below 0.05 were marked in italic

## Discussion

The principle steps in the diagnosis and treatment of esotropia are to determine the full amount of hyperopia and whether hyperopic correction will correct the esotropia [7]. Atropine sulfate, cyclopentolate, tropicamide, and mixed eye drops are widely used as cycloplegic agents [2–5]. Atropine is generally considered as a cycloplegic agent in hyperopic patients with dark pigmented irises [2, 3]. However, atropine obtains a maximal cycloplegic effect after approximately 3 days and recovery from cycloplegia takes 6 to 12 days. Atropine is also known for its possible systemic side effects including flushing, fever, and delirium [1]. Therefore, it should be avoided in children with heart problems or fever and those younger than 1 year. In contrast, tropicamide achieves a maximal cycloplegic effect 30 min after the initial application that continues for 15 min. Even though tropicamide is not thought to be strong enough to prevent accommodation in young children, it is widely used in busy clinics because of its convenience and few side effects [4, 8]. Mixed eye drop containing 0.5% tropicamide and 0.5% phenylephrine is a commercially available substitute for 1% tropicamide. Although, several studies reported that addition of 0.5% phenylephrine to tropicamide enhances the cycloplegic effect [9], we have experienced some patients with esotropia who showed an increased angle of deviation after instilling mixed eye drops containing 0.5% tropicamide and 0.5% phenylephrine.

In this study, 13 patients (38.2%) showed an increased amount of esodeviation after cycloplegia compared to that measured before cycloplegia, whereas none of the patients showed decreased esodeviation. All patients who showed increased angle of deviation had accommodative esotropia.

Several hypotheses may explain the phenomenon of increased esodeviation under cycloplegia. First, a decreased in effort of fusional divergence may contribute to increased esodeviation under cycloplegia. In the early stage of accommodative esotropia, the patients showed straight eyes and crossed sometimes when the child was tired. Similarly, blurred vision under cycloplegia caused children to stop attempting fusion and as a result, esodeviation increased depending on the patient’s divergence fusional amplitude [10]. However, it is known that the fusional divergence amplitude is typically weak in patients with accommodative ET, measuring 2–8 PD [11] compared to the normal range of 6–10 PD [1]. Considering that our definition of increased angle of deviation was 10 PD or more and two patients showed a difference as high as 20 to 25 PD, other factors in addition to the divergence amplitude might contribute to increased esodeviation. Second, although several studies reported that Mydrin-P, the 0.5% tropicamide and 0.5% phenylephrine combination, is an acceptable and useful cycloplegic agent [9, 12], it would be insufficient to achieve complete accommodation paralysis in dark pigmented eyes, particularly in young patients who have great accommodation effort [3]. The children may attempt to accommodate to clear the blurred image after cycloplegia as they did to correct their hyperopia. If the accommodative capability is retained as a result of incomplete cycloplegia, it induces a certain amount of associated reflex convergence by excessive innervational stimulation of accommodative effort and esodeviation is therefore increased. This hypothesis is supported by our finding that a larger difference between MR and CR was the only risk factor for increased esodeviation in both univariable (OR = 4.72, *P* = 0.029) and multivariable (OR = 5.22, *P* = 0.047) analyses. Furthermore, some parents of esotropes often reported that their children sometimes showed larger deviations than their esodeviation presented in the clinic. In these children, their angle of deviation was increased after cycloplegia and their parents reported the angle of deviation presented with cycloplegia was what they had noticed. Instilling mixed eye drops containing 0.5% tropicamide and 0.5% phenylephrine may help to reveal the latent angle of deviation in patients with esotropia and hyperopia. In contrast to our study, another study reported that children with accommodative esotropia demonstrated reduced esodeviation under cycloplegia with 1% atropine depending on their accommodative portion [13]. Different cycloplegic agents can have various effects on angle of esodeviation.

There are several limitations in this study. Firstly, this study was retrospective in design and the sample size was small. Secondly, the AC/A ratio was not evaluated by the gradient method, although most of the children demonstrated a normal AC/A ratio by clinical evaluation of distance–near relationship except for one case with a high AC/A ratio. Thirdly, changes in the angle of deviation under strong cycloplegics such as atropine were not measured in this study. However, it would be necessary to look at the comparison between atropine and mixed cycloplegia in further controlled studies. Finally, long-term motor and sensory outcome were not evaluated in this study. Further controlled studies were needed to assess long-terms prognosis in patients with increased angle of deviation under cycloplegia. Nevertheless, to the best of our knowledge, this is the first study to report increased esodeviation under cycloplegia in patients with hyperopia and esotropia.

## Conclusion

Children with accommodative esotropia and hyperopia often showed an increased angle of deviation after instillation of 0.5% tropicamide and 0.5% phenylephrine. This phenomenon reminded the clinicians that cycloplegics can have a different effect on esodeviation and suggested that increased angle of esodeviation may help to reveal the latent deviation in some patients with hyperopia and esotropia, even though it needs further investigation to examine the hypothesis.

## Abbreviations

BCVA: Best corrected visual acuity
CI: Confidence interval
CR: Cycloplegic refraction
FAET: Fully refractive accommodative esotropia
IQR: Interquartile range
MR: Manifested refraction
NAET: Non-accommodative esotropia
OR: Odds ratio
PAET: Partially refractive accommodative esotropia
PD: Prism diopters
SE: Spherical equivalent
